# The Role of ACTH and Corticosteroids for Sepsis and Septic Shock: An Update

**DOI:** 10.3389/fendo.2016.00070

**Published:** 2016-06-20

**Authors:** Djillali Annane

**Affiliations:** ^1^General Intensive Care Unit, Raymond Poincaré Hospital (AP-HP), Garches, France; ^2^Laboratory of Infection and Inflammation, U1173, University of Versailles Saint-Quentin-en-Yvelines University, INSERM, Garches, France

**Keywords:** sepsis, nitric oxide, cytokines, hypothalamic–pituitary–adrenal axis, stress response

## Abstract

Sepsis is a common disorder associated with high morbidity and mortality. It is now defined as an abnormal host response to infection, resulting in life-threatening dysfunction of organs. There is evidence from *in vitro* and *in vivo* experiments in various animal models and in patients that endotoxin or sepsis may directly and indirectly alter the hypothalamic–pituitary–adrenal response to severe infection. These alterations may include necrosis or hemorrhage or inflammatory mediator-mediated decreased ACTH synthesis, steroidogenesis, cortisol delivery to tissues, clearance from plasma, and decreased sensitivity of tissues to cortisol. Disruption of the hypothalamic–pituitary–adrenal axis may translate in patients with sepsis into cardiovascular and other organ dysfunction, and eventually an increase in the risk of death. Exogenous administration of corticosteroids at moderate dose, i.e., <400 mg of hydrocortisone or equivalent for >96 h, may help reversing sepsis-associated shock and organ dysfunction. Corticosteroids may also shorten the duration of stay in the ICU. Except for increased blood glucose and sodium levels, treatment with corticosteroids was rather well tolerated in the context of clinical trials. The benefit of treatment on survival remains controversial. Based on available randomized controlled trials, the likelihood of survival benefit is greater in septic shock versus sepsis patients, in sepsis with acute respiratory distress syndrome or with community-acquired pneumonia versus patients without these conditions, and in patients with a blunted cortisol response to 250 μg of ACTH test versus those with normal response.

Sepsis places a burden on health-care systems worldwide due to an annual incidence of about 100 per 100,000 inhabitants (1) and mortality rates between 15 and 40% (when shock is present) in the short term and up to 80% at 5 years (2). Moreover, roughly half of survivors may present with progressive decline in cognitive function (2, 3).

Sepsis is defined as an abnormal host response to infection, resulting in life-threatening dysfunction of organs (4). Host response to stress was originally described by Selye (5). The so-called general adaptation syndrome typically includes an early alarm phase, followed by a phase of resistance, and then a phase of exhaustion, which may result in death. Host response to stress relies on three major systems: the hypothalamic–pituitary–adrenal (HPA) axis, the autonomic nervous system, and the immune system (6). A correct balance between activation of these systems allows controlling infection while maintaining cardiovascular and metabolic homeostasis. A typical neuroendocrine response to stress includes (i) immediate increased secretion of catecholamines from the sympathetic nervous system and adrenal medulla, release of corticotrophin-releasing hormone (CRH) and vasopressin from parvocellular neurons into the portal circulation, and secretion of oxytocin from the neural lobe of the pituitary, (ii) 5–10 s later, secretion of corticotrophin (ACTH) by anterior pituitary cells, (iii) followed a few seconds later by decreased secretion of pituitary gonadotropins and increased secretion of prolactin and growth hormone (in primates), and of renin and glucagon from the kidneys and pancreas, respectively, and (iv) a few minutes later, increased plasma levels of glucocorticoids and inhibition of gonadal steroids secretion. Any imbalance between neuroendocrine and immune responses favoring a proinflammatory state may trigger organ dysfunction and progression of infection to sepsis.

This review will summarize current knowledge on HPA axis and disruption during sepsis and the potential role of treatment with corticosteroids.

## Activation of the Hypothalamic–Pituitary–Adrenal Axis during Sepsis

During stress, the HPA axis is mainly activated by CRH-independent pathways, involving immune mediators. The hypothalamus and pituitary glands are protected from exogenous or endogenous toxic molecules by the blood–brain barrier (BBB). Invading pathogens are identified by various cells, including epithelial, endothelial, and immune cells, thanks to danger molecule associated patterns (DAMP) they express on their surface or cytosol (7). Subsequently, these cells produce factors promoting recruitment of additional cells and destruction and clearance of pathogens. Among them, proinflammatory cytokines, such as tumor necrosis factor (TNF), interleukin (IL)-1 and -6, and anti-inflammatory cytokines, such as IL-4 and -10, may contribute activating the HPA axis.

### Activation of the Hypothalamic–Pituitary Axis

#### At the Hypothalamic–Pituitary Axis Level

There are three main routes for immune mediators to reach the hypothalamus and/or the pituitary gland. First, terminal nerve endings of autonomic nervous afferent fibers express pathogens or DAMP and receptors for many mediators (8). Then, sensing pathogens or related DAMP in tissues results in hypothalamic signaling *via* autonomic nuclei in the brainstem, which have projections to the hypothalamus, for example, between the locus ceruleus and the arcuate nucleus, and other structures of the limbic system as well. Then, efferent fibers, particularly of the vagus nerve, contribute to the attenuation of inflammation and in resuming homeostasis (9). Corticotrophin-releasing hormone is released upon acetylcholine stimulation of muscarinic receptor, an effect that is prevented by non-specific nitric oxide (NO) blockade (10). Second, inflammatory mediators released in blood from tissues can reach the portal circulation in the median eminence, located outside the BBB, *via* the anterior hypophyseal arteries. They are carried onto the brain structures, expressing receptors for these mediators, either through areas lacking a BBB, i.e., the circumventricular organs or across it using specific transporters (11, 12). Third, systemic inflammation may cause breakdown to the BBB, facilitating blood-borne cytokines traffic to deep brain structures (13–16). Among the various factors that contribute to the disruption of tight junctions or swelling of the BBB, the complement system, particularly C5a anaphylatoxin expressed both by astrocytes and endothelial cells, may play a key role (17).

Dendritic and microglial cells may produce immune molecules. In animals, peripheral administration of endotoxin yielded expression of IL-1 (18) and TNF (19). Similarly, in patients with septic shock, postmortem examination suggested overexpression of IL-1 and TNF in hypothalamic nuclei (20). Different cytokines in different brain regions induce different brain responses. For example, IL-1 and TNF are likely the two main mediators of the so-called sickness behavior, whereas IL-6 may have no apparent direct effect on behavior (21). Experiments in animals suggest that TNF- and IL-1-induced release of corticosterone is CRH-dependent mechanism (22, 23), whereas IL-6 may stimulate adrenal function by both CRH-dependent and -independent mechanisms (24). IL-1-related activation of the HPA axis is mainly dependent on brain endothelial cells and is independent of hematopoietic cells and perivascular macrophages (25).

#### At the Adrenal Gland Level

Tumor necrosis factor is produced in adrenal tissues by resident macrophages and by adrenocortical cells, particularly in the fasciculate and reticular layers (26). The presence within the adrenals of TNF and of its receptors suggests that this cytokine plays a role in adrenal function, even though experiments found variably stimulatory (27, 28) or inhibitory (26, 29) effects of TNF on steroidogenesis. Similarly, IL-1 and its receptor are also produced in adrenal tissues and may contribute to steroidogenesis at least partly by regulating prostaglandins pathways (30). Toll-like receptors (TLR) types 2 and 4 are expressed in human’s adrenal cortex (31). TLR2 or TLR4 knockout mice showed impaired glucocorticoid response to LPS (32, 33). Recent data suggested that these DAMP molecules expressed by immune cells recruited in adrenal tissues play a major role in the local immune-adrenal crosstalk (34).

### Mechanisms of Disrupted Hypothalamic–Pituitary–Adrenal Axis in Sepsis

#### Irreversible Damage to Neuroendocrine Cells

Sepsis is infrequently associated with necrosis or hemorrhage within the HPA axis. The venous drainage of the adrenals being limited, sepsis-associated massive increase in arterial blood flow to these glands results in enlarged glands (Table 1) (35). Then, adrenal necrosis and hemorrhage have been reported as a consequence of sepsis for more than a century (36, 37). Predisposing factors of the Waterhouse–Friderichsen syndrome include renal failure, disseminated intravascular coagulopathy, and treatment with anticoagulants or tyrosine kinase inhibitors. Ischemic lesions and hemorrhage have also been described within the hypothalamus or pituitary gland (38).

**Table 1:** **Mechanism explaining hypothalamic–pituitary–adrenal axis disruption in sepsis**.

HPA axis level	Main mechanisms	Precipitating factors
Hypothalamus	Necrosis or hemorrhage	Anticoagulants, brisk variations in blood pressure, high dose of vasopressors
		Coagulopathy, severe hypoxia, hyperglycemia
	Decreased CRH/AVP synthesis/release	Treatment with corticosteroids, psychoactive drugs
		Increased brain levels of proinflammatory cytokines (mainly TNF and IL-1)
		Hypercortisolemia
Pituitary gland	Necrosis or hemorrhage	Anticoagulants, brisk variations in blood pressure, high dose of vasopressors
		Coagulopathy, severe hypoxia, hyperglycemia
	Decreased ACTH synthesis/release	Treatment with corticosteroids, psychoactive drugs, anti-infective drugs, megestrol acetate medroxyprogesterone
		Increased blood levels of proinflammatory cytokines (mainly TNF and IL-1)
		Coagulopathy, severe hypoxia, hypercortisolemia
Adrenals	Necrosis or hemorrhage	Anticoagulants, brisk variations in blood pressure, high dose of vasopressors
		Coagulopathy, severe hypoxia
	Decreased steroidogenesis	
	Depletion of lipid droplets	Cholesterol-lowering drugs
	Decreased expression of scavenger receptor B1	Proinflammatory mediators
	Enzymes inhibition	Aminoglutethimide, ketoconazole, fluconazole, etomidate, dexmedetomidine
		Proinflammatory mediators
	Decreased sensitivity of ACTH receptors	Circulating and adrenals proinflammatory mediators (e.g., corticostatins)
Tissue resistances	Decreased cortisol delivery to tissues	Proinflammatory mediators, liver failure, severe denutrition
	Accelerated glucose clearance	Phenobarbital, phenytoin, rifampin
	Decreased binding capacity and affinity of glucocorticoid receptor	Proinflammatory mediators

*HPA, hypothalamic–pituitary–adrenal*.

#### Altered CRH/ACTH Synthesis

Hypothalamic/pituitary stimulation by cytokines, particularly IL-1, induced a biphasic response with initial proportional increase followed by progressive decline in anterior pituitary ACTH concentrations (39, 40). Sepsis is associated in animals (41, 42) and in humans (20) with marked overexpression of the inducible isoform of NO synthase (iNOS) in hypothalamic nuclei that is partly triggered by TNF and IL-1. Subsequent abundant release of NO may cause apoptosis of neurons and glial cells in the neighborhood. In both rodents and humans, sepsis decreased ACTH synthesis, though its secretagogues remained unaltered (43). Then, the suppression in ACTH synthesis following sepsis may be mediated by NO (11).

ACTH synthesis can also be inhibited by various treatments (44). Opioids are the main component of patients’ sedation regimen in ICU worldwide. In animals, depending on dose, timing, and duration, opioids have been shown to variably stimulate or inhibit the CRH/ACTH axis, whereas in humans, they predominantly inhibited it (45). In animals, sepsis is associated with early marked increase in ACTH levels that returned to baseline values around 72 h (46). Clinical studies have found ACTH levels to be significantly lower in critically ill patients (47, 48) and particularly in septic shock (48) than in controls. However, altered ACTH synthesis in response to metyrapone was observed in roughly half of septic shock, and very occasionally in patients without sepsis (48).

#### Altered Steroidogenesis

The adrenals storage of cortisol is very limited. Therefore, adequate response to stress relies almost entirely on cortisol synthesis. The normal HPA axis response to sepsis remains unknown. Cortisol production rate is increased in critically ill patients (47). The main finding in this study was an average 50% reduction in cortisol clearance from plasma, mainly resulting from a loss in cortisol inactivation through suppressed liver and renal cortisol to cortisone shuttle. About half of septic-shock patients have decreased cortisol synthesis (48). Following administration of metyrapone, 60% of septic shock had 11β deoxycortisol concentrations <7 μg/dl, suggesting decreased cortisol synthesis. The alteration may occur at various steps in the cortisol synthesis chain. First, histological examination of the adrenal cortex of both animals and humans with sepsis found marked depletion in lipid droplets, suggesting deficiency in esterified cholesterol storage (49). This loss in lipid droplets is likely mediated by annexin A1 and formyl peptide receptors (50). In normal conditions, both increased plasma ACTH concentrations and depletion in adrenal cholesterol stores upregulate adrenals scavenger receptor B1 (SRB1), an HDL receptor, which captures esterified cholesterol from blood (51). SRB1-mediated cholesterol uptake is considered an essential protective mechanism against endotoxin (52). Then, sepsis-induced deficiency, in SRB1 expression by the adrenal cortex, was associated with increased mortality (53). Second, a number of environmental factors may inhibit adrenal steroidogenesis (54). Steroidogenesis may be inhibited at various enzymatic steps by drugs, including P-450 aromatase, hydroxysteroid dehydrogenase, or mitochondrial cytochrome P-450-dependent enzymes (44). In critically ill patients, etomidate, which inhibits the last enzymatic step in cortisol synthesis, increased the risk of adrenal insufficiency, 4–6 h (OR 19.98; 95% CI 3.95–101.11) and 12 h (OR 2.37; 95% CI 1.61–3.47) post-dosing (55). This effect was associated with organ dysfunction, but the ultimate effects on mortality remained unclear. Finally, inflammatory mediators, such as corticostatins, may bind to ACTH receptors in the adrenal cortex, thus preventing ACTH stimulation of cortisol synthesis (56).

#### Tissues Resistance to Glucocorticoids

A number of factors may prevent cortisol bioactivity in tissues. First, cortisol clearance may be accelerated, particularly following administration of various drugs, for example, psychoactive drugs (barbiturates, phenytoin) or antibiotics (rifampicin) (52). Second, sepsis is often associated with marked reduction in corticosteroid-binding globulin (CBG) and albumin (48, 57). On the one hand, the reduction in cortisol carriers increased free cortisol concentrations in plasma. On the other hand, cortisol bound to CBG is specifically released at the level of inflamed tissues, *via* neutrophils elastase-dependent mechanisms (58, 59). Thus, the net effect of sepsis-associated reduced CBG and albumin levels is reduced cortisol delivery to local sites of inflammation. Third, at tissue levels, T-helper 2 cell-derived cytokines, for example, IL-2 or IL-4, may inactivate cortisol to cortisone by upregulating the 11β-hydroxysteroid dehydrogenase (11β-HSD) type 2 enzyme (60). Finally, downregulation of the glucocorticoid receptor (GR)-α is a well-known complication of sepsis (61). The decrease in GR binding and affinity may be at least partly related to exaggerated release of NO in tissues (62). Sepsis may also cause alteration in the translocation of the GR-α (63). The loss in the dimerization of the GR-α caused resistance to glucocorticoids and lethality in septic animals (64).

## Corticosteroids for Sepsis and Septic Shock

Corticosteroids have been used for more than 60 years in the management of patients with severe infections. There is a strong rationale (as described earlier) for exogenous administration of glucocorticoids in sepsis. Nevertheless, their use in practice still remains controversial. There is a general agreement that corticosteroids improve sepsis-associated comorbidities, such as shock, organ dysfunction, and length of hospital stay. Their effects on survival and on the risk of secondary infections are controversial.

### Corticosteroids Improve Cardiovascular Function

Corticosteroids contribute to restoring effective blood volume, notably *via* sodium and water retention by binding to mineralocorticoid receptors in the kidney. They also contribute to restoring systemic vascular resistance. First, increase in sodium and water content in a vessel’s interstitium results in increased stiffness of the vessel wall. Second, corticosteroids enhance vascular contractile (65) and blood pressure (66) responses to α-1 agonists. This effect occurs within minutes following corticosteroid administration and is likely a non-genomic effect *via* modulation of the α-1 agonists’ receptors second messenger (65) and ATP-sensitive potassium channels (67). The endothelial GR is crucial for preventing prolonged activation of NO and NF-κB, following sepsis (68). Thus, prolonged improvement in vascular responsiveness to corticosteroids is likely a genomic transrepressive effect. Patients with septic shock and blunted response to 250 μg ACTH bolus, i.e., increase in total cortisol of <9 μg/dl, have more depressed systemic vascular resistance and a greater effect of hydrocortisone bolus on blood pressure response to norepinephrine than patients with intact HPA axis (65). Corticosteroids also improved microcirculation and tissue perfusion in septic shock (69). This effect may be mediated by upregulation of endothelial NO synthase *via* activation of the mitogen-activated protein kinase and protein Akt pathway (70).

A recent systematic review found 12 trials (*n* = 1561 patient) and reported the effects of corticosteroids on shock reversal (weaned off vasopressor therapy) by 1 week (71). In this review, the relative risk (RR) of having shock reversed by day 7 was 1.31 (95% CI 1.14–1.51; *P* value = 0.0001, random effects model), in favor of corticosteroids.

### Corticosteroids Decrease Organ Failure

There is strong evidence that corticosteroids attenuate inflammation in various organs in sepsis. For example, they have been shown to dramatically decrease NF-κB activity in peripheral immune cells (72) or in the lung (73). Corticosteroids have been shown to inhibit iNOS activation in the renal cortex, preventing hypoxic injuries and restoring an adequate oxygen delivery to oxygen balance (74). They also improve glomerular function (75), free water clearance, and sodium renal excretion (76). Corticosteroids may attenuate sepsis-associated brain inflammation particularly by preventing the breakdown of the BBB (77, 78). A total of eight trials (*n* = 1132 patients) (71) reported a dramatic reduction in the number and degree of severity of failing organs, with a mean reduction in the SOFA score – a measure of organ dysfunction (79) – of −1.53 (−2.04 to −1.03; *P* value < 0.00001), in favor of corticosteroids. Corticosteroids also reduced ICU length of stay by −1.68 days (−3.27 to −0.09; *P* value = 0.04) and −2.19 days (95% CI −3.93 to −0.46; *P* value = 0.01), in ICU survivors (71).

### Corticosteroid Tolerance

#### Secondary Infections

Corticosteroids shift the recruitment of T cells from T-helper type 1 to T-helper type 2 and thus to favor the production of anti-inflammatory cytokines. Data from 19 trials (*n* = 2567 patients) found that the RR for superinfection was 1.02 (0.87–1.20; *P* value = 0.81) (71). Corticosteroids may blunt febrile response to infection and alter leukocyte count and most inflammatory biomarkers. Thus, it may become difficult to recognize secondary infections in corticosteroid-treated patients. In practice, physicians should systematically screen on a daily basis any potential source of infection and draw samples for bacterial culture.

#### Metabolic Complications

Corticosteroids induce hyperglycemia by stimulating neoglucogenesis, glycogenolysis, and by insulin resistance in skeletal muscles and adipocytes. In septic shock, corticosteroids are associated with hyperglycemia (*P* value < 0.00001) and hypernatremia (*P* value < 0.00001) (71). As compared with bolus administration, continuous infusion of corticosteroids may ease the control of glycemia in sepsis (79). However, preventing hyperglycemia by intensive insulin therapy did not improve morbidity or mortality (80).

#### Acquired Neuromyopathy

Myopathy is a common complication of prolonged or acute exposure to corticosteroids, particularly high doses of fluorinated derivatives (e.g., dexamethasone). They induce myonecrosis, diffuse atrophy of fibers, cumulated sarcoplasmic glycogen vesicles, myofibril disorganization, and selective depletion of thick myosin filaments (81, 82). Upregulation of the calpain pathway suggests that altered calcium metabolism and/or increased proteolysis may contribute to corticosteroid muscle toxicity (82). The risk of myopathy associated with corticosteroids may be potentiated by hyperglycemia, hypoxia, or non-depolarizing neuromuscular drugs.

#### Other Complications

In theory, corticosteroids may be associated with psychotic disorders or gastroduodenal bleeding. In practice, data from 19 trials (*n* = 2382) found that the RR of gastroduodenal bleeding was of 1.24 (95% CI 0.92–1.67; *P* value = 0.15) (71). ICU studies variably found that exposure to systemic corticosteroids increased (83) or not (84) the risk of transition to delirium. Corticosteroid weaning may be associated with psychiatric manifestations, including depressive state and apathy.

### Corticosteroids’ Effects on Survival

Most experiments, in both small and large animals, based on endotoxin challenges or live bacteria-induced sepsis, found survival benefit from various doses and durations of corticosteroids (85). At least 33 trials have evaluated corticosteroids for severe infection with or without septic shock (71). Data from 27 trials (*n* = 3176 patients) found a RR of dying at 28 days of 0.87 (0.76–1.00, *P* value = 0.05). The survival benefit was more remarkable (*P* value = 0.01) in 22 trials of prolonged (>96 h) treatment with a moderate (<400 mg daily of hydrocortisone or equivalent) dose of corticosteroids. In this Cochrane review, meta-regression found a significant dose effect of corticosteroids, i.e., the lower the dose, the lower the RR of dying. This review also suggested that septic shock, ARDS, or community-acquired pneumonia were more likely to draw a survival benefit. Finally, data from eight trials (*n* = 583 patients), reporting subgroups based on the response to 250 μg ACTH test, found a RR of dying of 0.88 (0.88–1.02, *P* value = 0.09), in favor of corticosteroids. Depending on trial selection and definition of outcomes, different meta-analyses variably found (86), or did not find (87, 88), survival benefit from corticosteroids. Current international guidelines recommend restricting the use of hydrocortisone to vasopressor-dependent septic shock (89).

## Conclusion

There are numerous experimental and clinical data establishing the paramount importance of an appropriate activation of the HPA axis to respond to severe infection. Similarly, experiments in animals and clinical observations strongly support the role of an inadequate HPA axis response in the physiopathology and outcome of sepsis. In most animal studies, corticosteroid administration consistently protected against lethal sepsis. In contrast, clinical trials in sepsis found much less consistency in survival benefits from corticosteroids, though most trials demonstrated faster resolution in shock and organ dysfunction. Thus, physicians should consider corticosteroids mainly in septic shock who do not respond rapidly to fluid therapy and vasopressors. Trials also consistently found that corticosteroids should be given at doses of 200 mg of hydrocortisone equivalent per day for at least 3 days at full dose.

## Author Contributions

DA is the sole author and is responsible for the whole content.

## Conflict of Interest Statement

The author declares that the research was conducted in the absence of any commercial or financial relationships that could be construed as a potential conflict of interest.
